# Assessment of Body Posture of Children With Chest Pain

**DOI:** 10.3389/fped.2021.704087

**Published:** 2021-08-17

**Authors:** Anna Zmyślna, Arkadiusz Łukasz Żurawski, Grzegorz Śliwiński, Zbigniew Włodzimierz Śliwiński, Wojciech Piotr Kiebzak

**Affiliations:** ^1^Collegium Medicum, Department of Health Sciences, Jan Kochanowski University, Kielce, Poland; ^2^Centre for Pediatrics, Regional Hospital in Kielce, Kielce, Poland; ^3^Faculty of Electrical and Computer Engineering, Institute of Biomedical Engineering, Technische Universität Dresden, Dresden, Germany

**Keywords:** posture, chest pain, children, musculoskeletal disorders, photogrametric measurement

## Abstract

**Objective:** An increase in the appearance of chest pain among children is observed globally. The authors present various reasons for their appearance. As can be seen from numerous observations, the majority of cases are not related to the pathology of the circulatory system. Increasingly, studies on the causes of chest pain in children show their association with musculoskeletal disorders.

**Aim:** of the work was assessment of body posture in children with chest pain using the Diers Formetric 4D system.

**Methods:** The study involved a group of 184 female and male children, aged 7–12 years. The study group consisted of 64 patients with chest pain. The children from this group were diagnosed with functional chest pain by a cardiologist. The control group consisted of 120 patients without chest pain. The study included the assessment of body posture using the DIERS Formetric system.

**Results:** The analysis of the results obtained during the study showed that among the children with chest pain, there are statistically significant irregularities in the parameters determining body posture compared to the control group.

Comparing the study group with the control group, there is a statistically significant difference in the lateral deviation of VPDM (rms) (mm) (*p* = 0.001). Both children from the test group aged 9–10 and 11–12 obtained higher results than their peers from the control group. In the group of the youngest children in terms of the lateral deviation of VPDM (rms) (mm), increasing the number of children under study would contribute to significant differences in this variable.

In the study group, among children aged 9–10 years, there were also statistically significant abnormalities regarding trunk imbalance and pelvic skewness compared to the children of the same age in the control group.

**Conclusions:** Irregularities in the parameters determining body posture may cause chest pain in children.

## Introduction

An increase in the appearance of chest pain among children is observed globally. The authors present various reasons for this. Musculoskeletal, gastrointestinal, pulmonary, idiopathic and psychogenic disorders are emphasized (1–5). As can be seen from numerous observations, the majority of cases are not related to the pathology of the circulatory system (6). In the study on the percentage of children referred due to chest pain to the University of Wisconsin/Children's Wisconsin, Herma Heart Institute, pediatric cardiac care center, Hanson stated that in 95% of patients the pain was not of cardiac origin (7). Skieska et al. also noted that out of 109 children complaining of chest pain, only 5.5% had cardiological etiology (8). Similar observations were made by Cohn et al. who stated that only 5% of chest pain in children and adolescents was related to cardiological problems (9).

Increasingly, studies on the causes of chest pain in children show their association with musculoskeletal disorders (10–14). Aygun et al. noted that the most prevalent causes of chest pain among 782 patients aged between 3 and 18 years were disorders of the musculoskeletal system (33%) (15). Similar observations were made by Sert et al. who stated that out of 380 children complaining of chest pain, 37%, had disorders of the musculoskeletal system (1).

There are works in the literature linking the appearance of chest pain with abnormal body posture (4).

According to the literature, there are many definitions of body posture. Some researchers consider body posture to be an alignment of chosen body parts in a relaxed vertical position. Maintaining a good body posture is related to its stability and balance (16, 17). The American Academy of Orthopedic Surgeons emphasizes that the balance between the muscle and bone tissue affects the correct body posture and protects the body against injuries (18). Kasperczyk interprets body posture as an optimal system of body sections enabling the achievement of maximum stability with little muscle involvement. This system creates suitable conditions for internal organs (19).

Body posture is shaped throughout life and depends on both internal and external factors. Age, hobbies, but also emotional state may influence the change of posture (20). A variety of body types and changeability of posture make it difficult to visually assess body posture. For this reason, in the assessment of body posture, objective research tools are increasingly used in order to obtain reliable data. This affects the reduction of the risk of making a mistake when making a diagnosis or planning the improvement procedure, but also the achievement of better therapy results. Despite numerous data on the causes of chest pain associated with musculoskeletal disorders, the literature lacks scientific reports on the objective and visual assessment of body posture in children with chest pain complaints (11, 20–22).

### Aim of the Work

Assessment of body posture in children with chest pain using the Diers Formetric 4D system.

## Materials and Methods

### Study Material

The study included all children who came to the clinic during the project period and qualified to participate in the experiment according to the inclusion criteria.

The study involved a group of 184 female and male children, aged 7–12 years. The children from the study and control groups were divided into age groups: 7–8, 9–10, and 11–12 years, in order to standardize the groups in terms of anthropometric indicators and the stage of body posture development.

The study group consisted of 64 patients with chest pain of the Department of Physiotherapy of the Swietokrzyskie Pediatrics Center in Kielce. The children from this group were diagnosed with functional chest pain by a cardiologist. The study group included 14 children aged 7–8 years, 20 children aged 9–10 years and 30 children aged 11–12 years.

The control group consisted of 120 patients without chest pain, of the Physiotherapy Department of the Swietokrzyskie Pediatrics Center in Kielce. The control group consisted of 14 children aged 7–8 years, 33 children aged 9–10 years and 73 children aged 11–12 years.

The study group consisted of 33 girls and 31 boys, while the control group consisted of 64 girls and 56 boys.

#### Inclusion Criteria

- age 7–12 years;
- a referral from a cardiology clinic to the Physiotherapy Department for a child with chest pain and diagnosis R29.8 acc. to ICD 10, denoting other and unspecified disease symptoms related to the nervous and musculoskeletal systems;
- no comorbidities that could affect the axis of the body;
- consent of the legal guardian to participate in the study.

#### Exclusion Criteria

- presence of comorbidities that may affect the axis of the body, such as: mental disorders, genetic diseases, scoliosis, abnormal structure of the chest;

### Study Method

**Figure 1:**
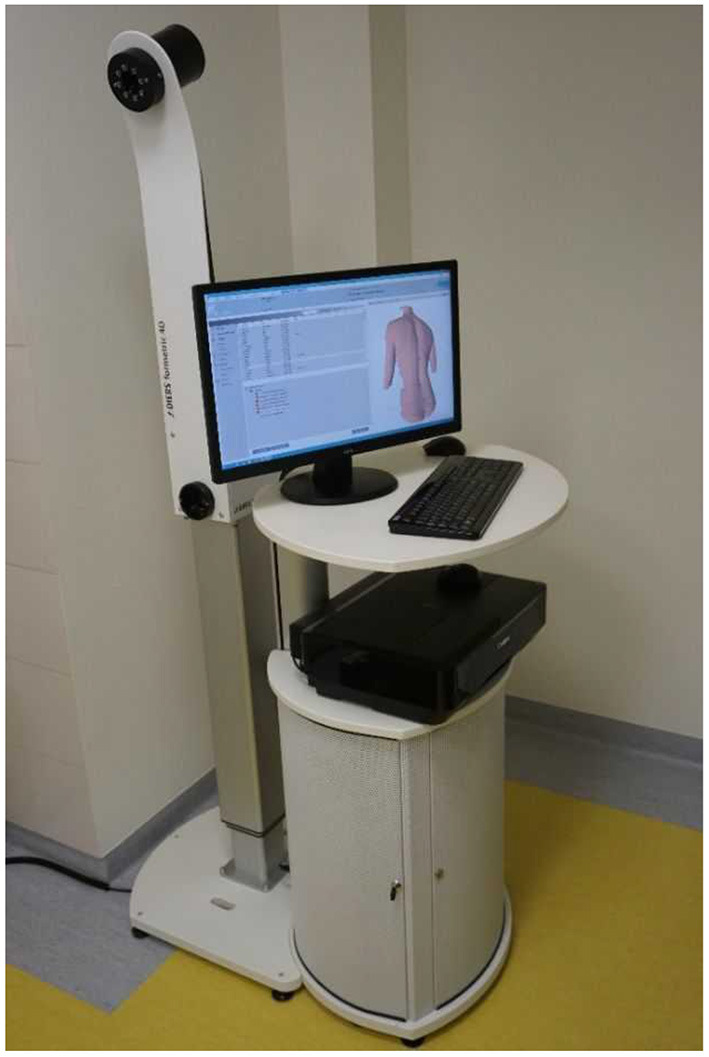
DIERS Formetric (own source).

The study included the assessment of body posture using the DIERS Formetric system. The system uses the method of surface topography, which enables the performance of a digital 3D reconstruction of the spine and scanning without radiation and without the use of markers. The system automatically detects anatomical landmarks such as the line of the spinous processes and the rotation of the vertebrae. It enables an objective and quantitative analysis of body posture using various clinical parameters (Figure 1) (23).

### The Course of the Examination

**Figure 2:**
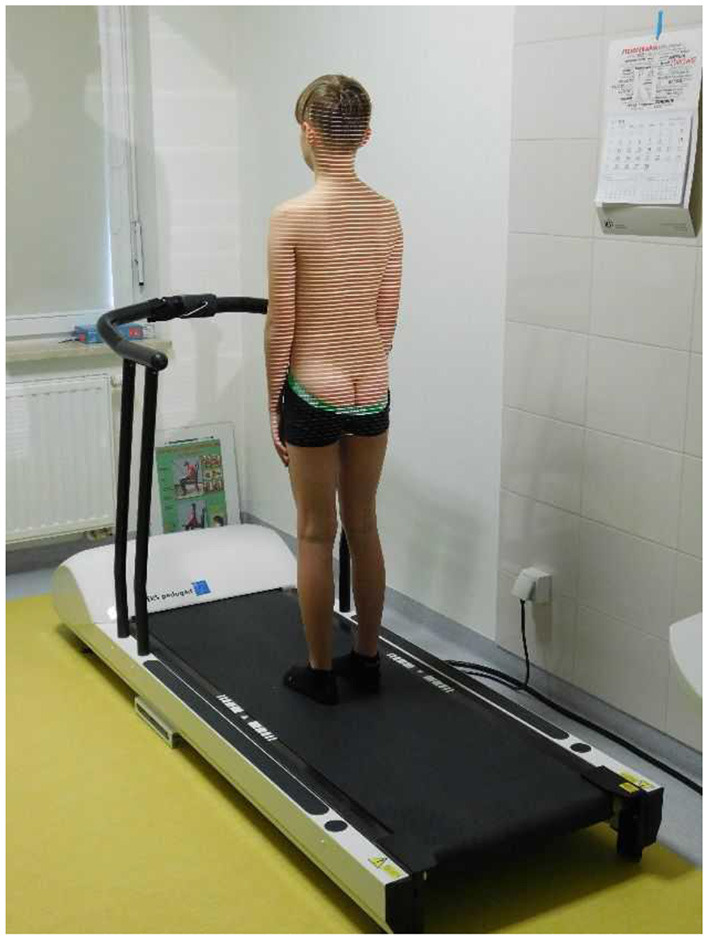
Examination of body posture with the DIERS Formetric 4D system (own source).

During the examination using the DIERS Formetric 4D, the patient was on the DIERS Pedoscan platform in a relaxed standing position, with his back to the examiner (Figure 2). In order to get an accurate measurement, the subject was asked to tie their hair if necessary and slide their underwear down to the intergluteal cleft. The patient was standing 2 meters away from the computer and the camera emitting light lines on the patient's back. The test room was darkened for optimal data recording. The camera was set at the height of the angles of the patient's lower shoulder blades. After the precise setting of the equipment and positioning of the examined person, an electronic record of the body posture and computer analysis followed. The examination with the DIERS Formetric 4D system included the assessment of 7 parameters, providing quantitative variables relevant for the statistical analysis. The examination using the DIERS Formetric 4D system was performed in both the study and control groups.

**Figure 3:**
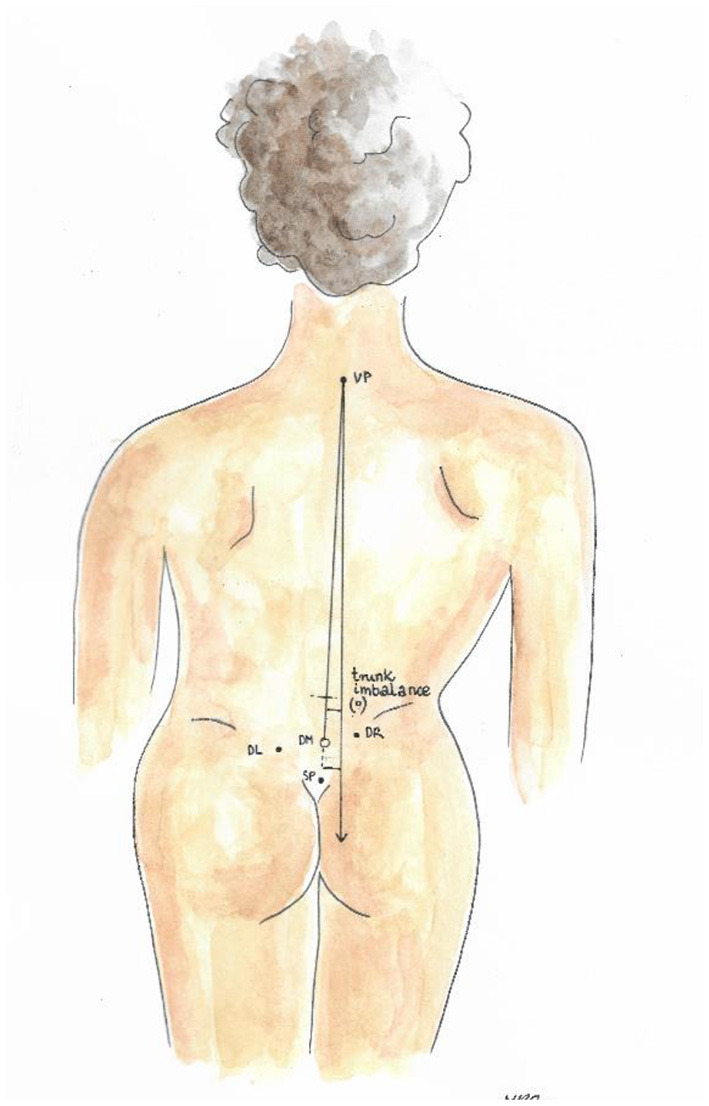
Trunk imbalance VP – DM.

**Figure 4:**
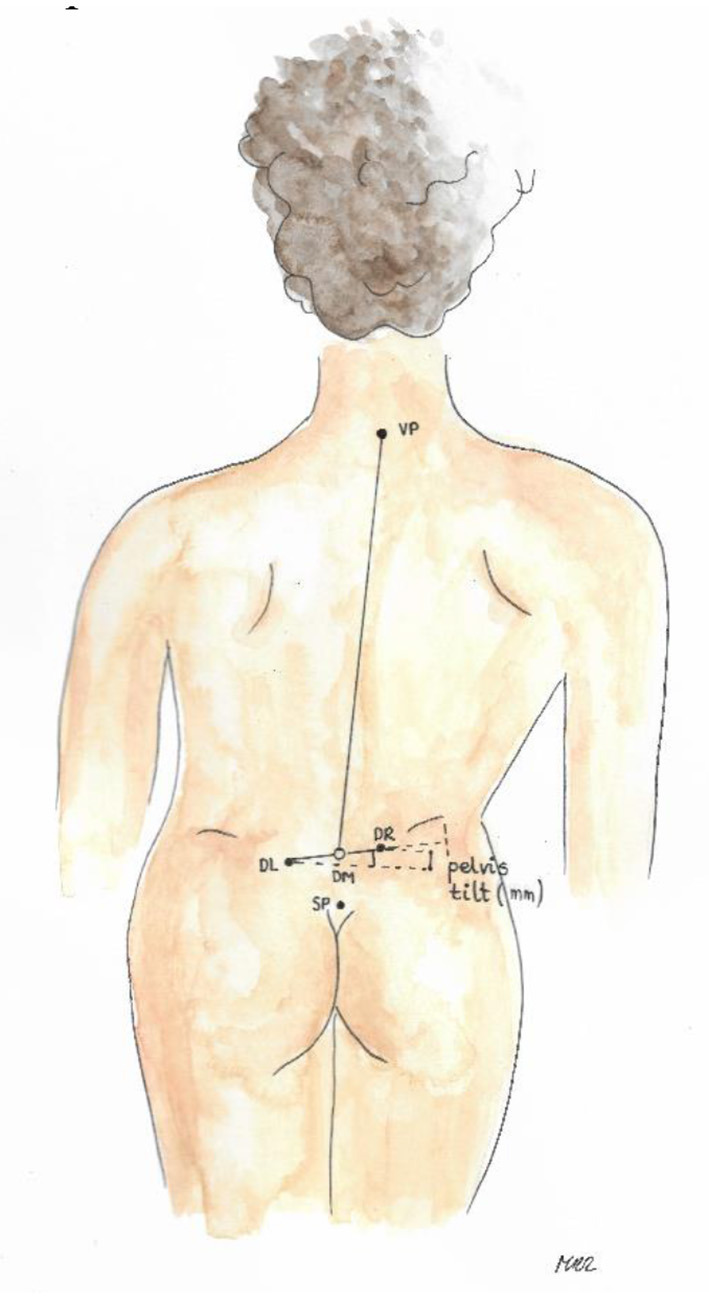
Pelvic skewness.

**Figure 5:**
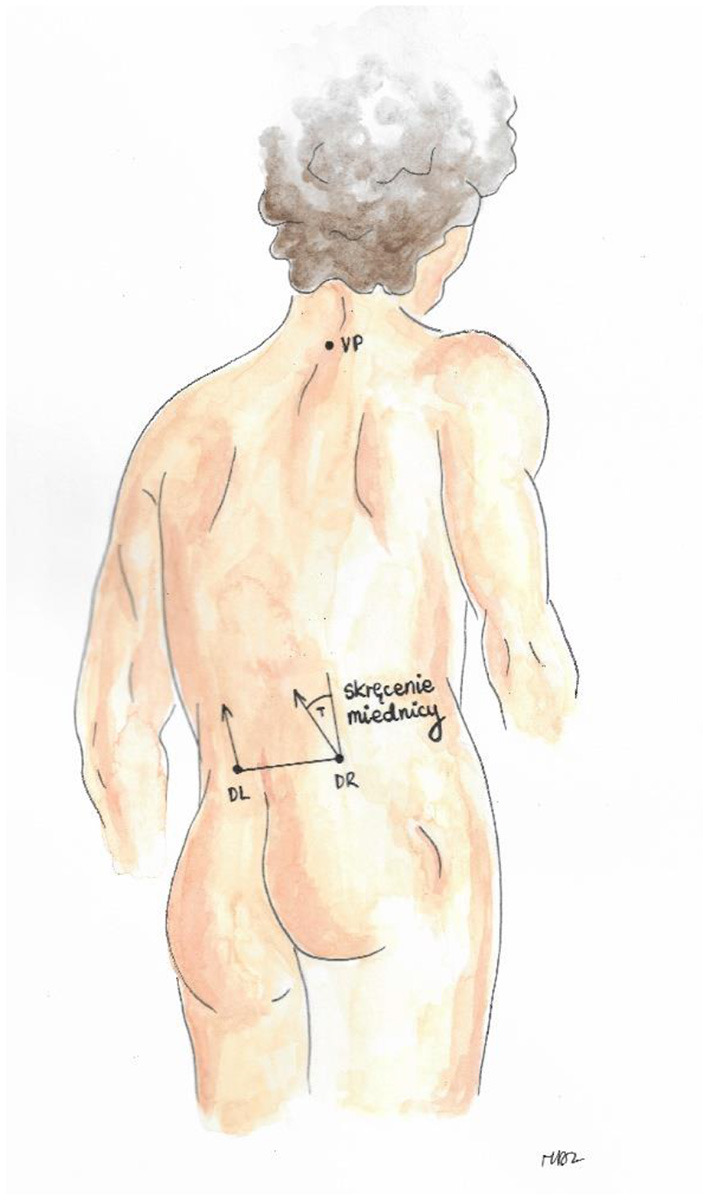
Pelvic torsion.

**Figure 6:**
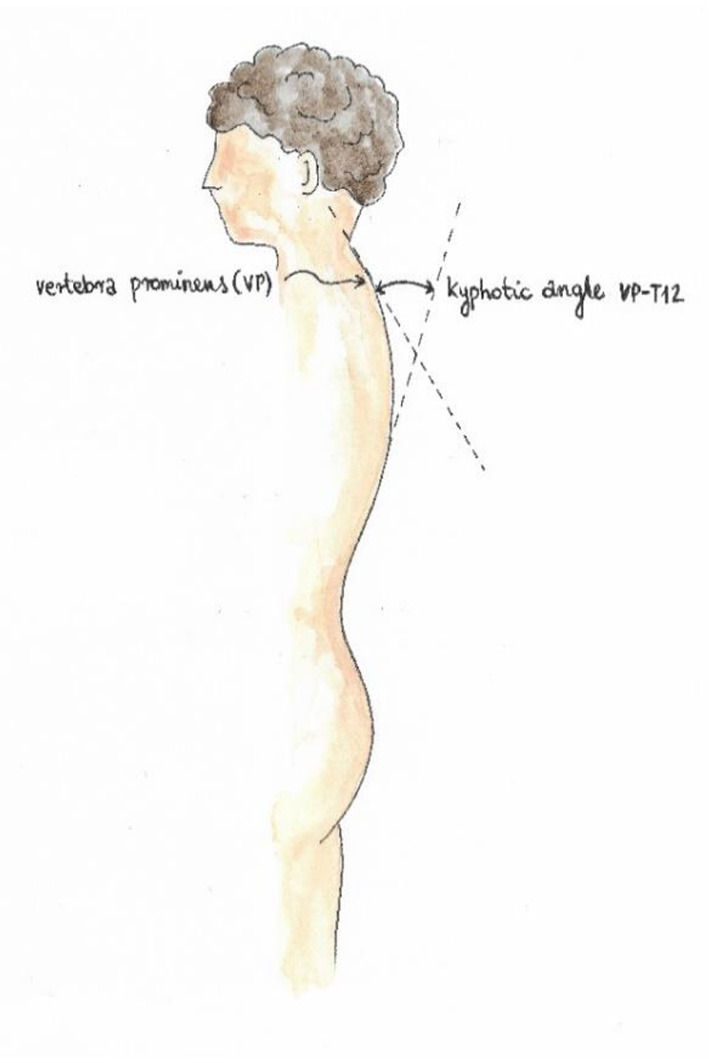
Kyphosis angle.

**Figure 7:**
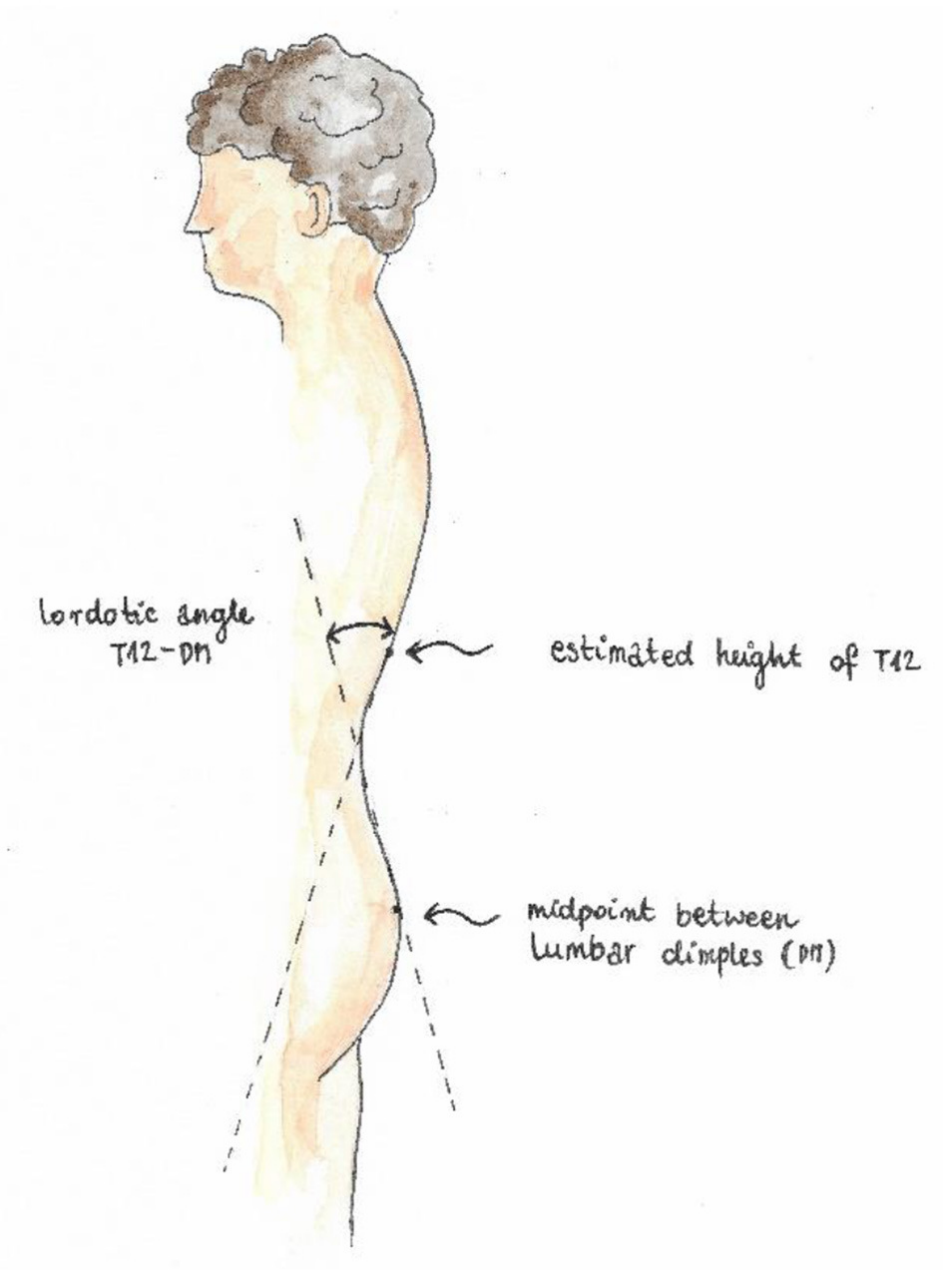
Lordosis angle.

**Figure 8:**
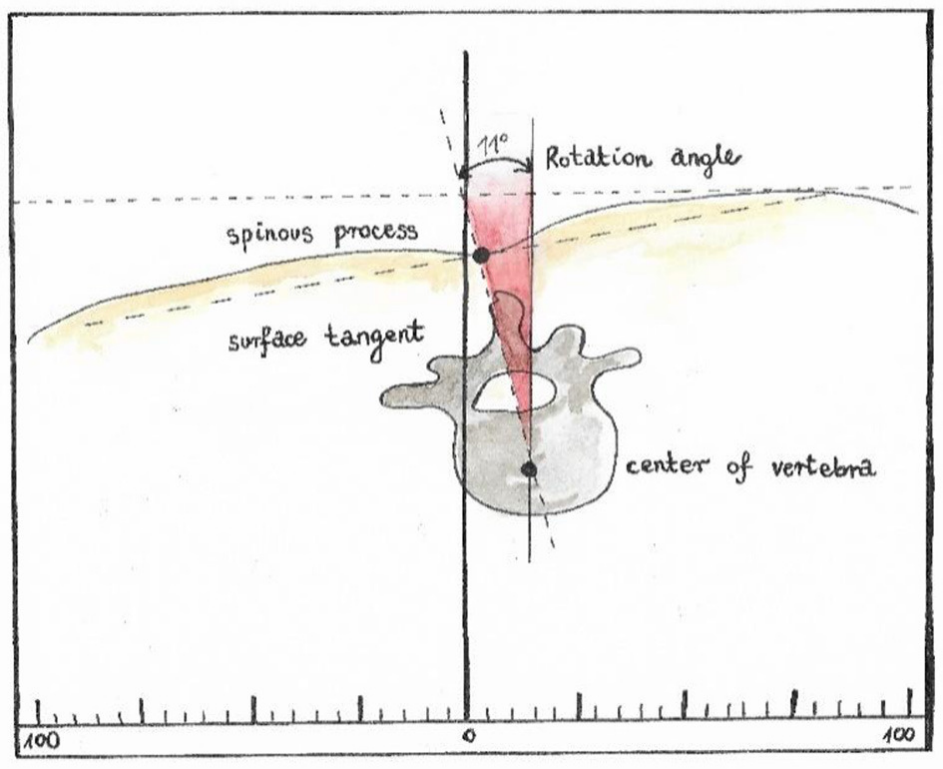
Surface rotation.

**Figure 9:**
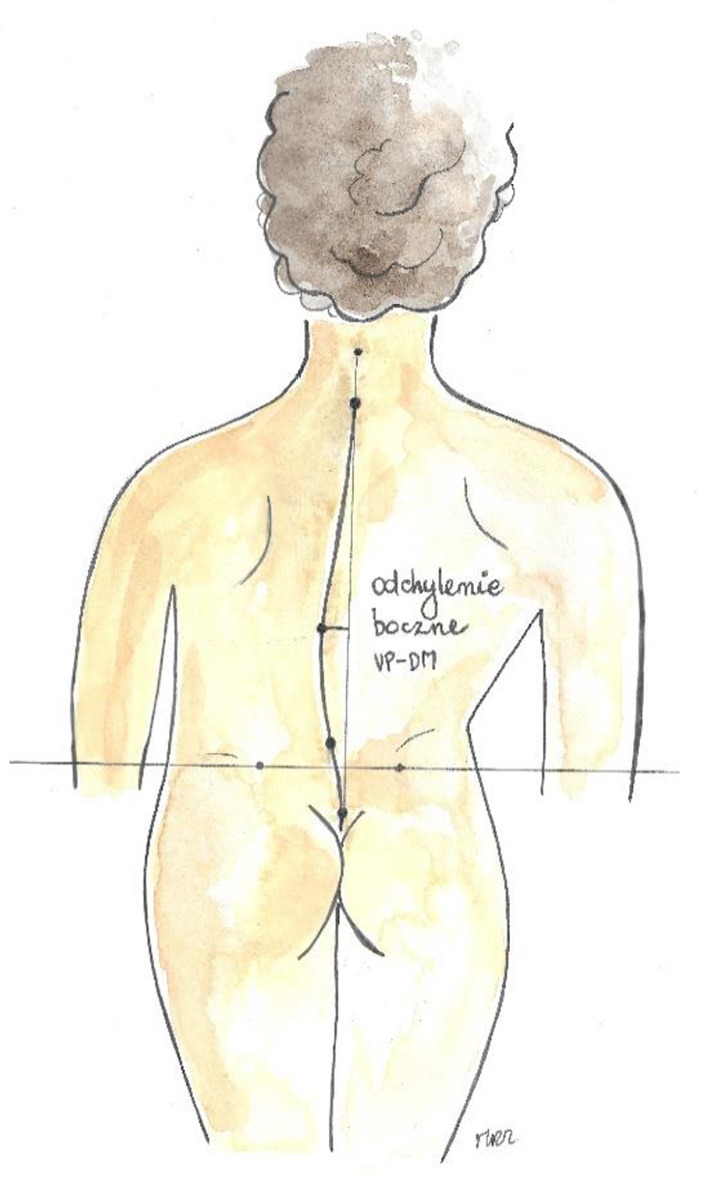
Lateral deviation (own source).

Parameters analyzed during the examination with the DIERS Formetric 4D system:

1. Trunk imbalance VP - DM: This parameter represents the deviation of the spinous process of the 7th cervical vertebra (VP) from the center of the line joining the right and left anterior superior iliac spine (DM). The value is measured in millimeters (Figure 3).
2. Pelvic skewness: This parameter shows the difference in the height of the dimples of Venus in the transverse plane. The value is measured in millimeters (Figure 4).
3. Pelvic torsion: This parameter is determined at the points of the dimples of Venus on the basis of the mutual torsion of the planes. It is measured in degrees (Figure 5).
4. Kyphosis angle: This parameter measures the angle between the spinous process of the 7th cervical vertebra (VP) and the estimated position of the spinous process of the 12th thoracic vertebra (Th12). The value is measured in degrees (Figure 6).
5. Lordosis angle: The parameter that determines the angle between the estimated position of the spinous process of the 12th thoracic vertebra (Th12) and the center of the line joining the right and left superior posterior iliac spines (DM). The value is measured in degrees (Figure 7).
6. Surface rotation: This parameter shows the maximum rotation of the surface on the symmetry line. The value is measured in degrees (Figure 8).
7. Lateral deviation VP - DM: This parameter shows maximum deviation of the midline of the spine from the VP-DM line in the frontal plane. It refers to the value at the top of curve arch. The parameter is measured in millimeters (Figure 9).

## Results

The average age in the study group was slightly above 10 years (M = 10.09; SD = 1.79), while in the control group it was almost 11 years (M = 10.71; SD = 1.47).

The average age among girls from the study group (M = 10.06; SD = 1.82) was comparable to the mean age among boys (M = 10.13; SD = 1.78). Similarly, among children from the control group, the mean age of girls (M = 10.72; SD = 1.37) was similar to the mean age of boys (M = 10.70; SD = 1.58).

The level of anthropometric indicators (height, weight, BMI) among the examined children. The analyzes were divided into subgroups, assuming the occurrence of pain, age and gender as the criterion of division (Tables 1–3).

**Table 1:** Anthropometric indicators among 7–8-year-old children depending on the occurrence of chest pain and gender.

**Children 7–8 years old**						
	**Girls from study group (** ***n*** **=** **7)**			**Boys from the study group (** ***n*** **=** **7)**		
	**height (m)**	**weight (kg)**	**BMI**	**height (m)**	**weight (kg)**	**BMI**
*M*	1.26	23.86	15.03	1.27	27.43	16.85
*SD*	0.05	3.76	2.25	1.27	2.40	1.69
*Me*	1.26	23.00	14.49	1.27	24.00	16.95
*Min*	1.18	19.00	12.56	1.19	22.00	13.82
*Maks*.	1.34	30.00	17.95	1.38	37.00	19.84
	**Girls from the control group (** ***n*** **=** **7)**			**Boys from the control group (** ***n*** **=** **7)**		
	**height (m)**	**weight (kg)**	**BMI**	**height (m)**	**weight (kg)**	**BMI**
*M*	1.32	35.57	20.07	1.25	32.43	20.59
*SD*	0.15	9.33	1.98	0.14	5.88	1.39
*Me*	1.32	32.00	19.07	1.20	30.00	20.83
*Min*	1.14	24.00	17.79	1.12	26.00	18.67
*Maks*.	1.53	48.00	22.52	1.50	42.00	22.32

**Table 2:** Anthropometric indicators among 9–10-year-old children depending on the occurrence of chest pain and gender.

**Children 9–10 years old**						
	**Girls from study group (** ***n*** **=** **11)**			**Boys from the study group (** ***n*** **=** **9)**		
	**height (m)**	**weight (kg)**	**BMI**	**height (m)**	**weight (kg)**	**BMI**
*M*	1.35	29.82	16.25	1.39	30.00	15.37
*SD*	0.09	6.82	2.57	0.08	4.21	1.16
*Me*	1.34	28.00	15.87	1.39	30.00	15.11
*Min*	1.26	22.00	13.43	1.28	24.00	13.70
*Maks*.	1.51	43.00	22.05	1.50	35.00	19.96
	**Girls from the control group (** ***n*** **=** **17)**			**Boys from the control group (** ***n*** **=** **16)**		
	**height (m)**	**weight (kg)**	**BMI**	**height (m)**	**weight (kg)**	**BMI**
*M*	1.40	39.24	19.91	1.42	42.56	21.08
*SD*	0.11	7.45	2.46	0.12	9.92	3.42
*Me*	1.40	39.00	18.90	1.44	40.50	20.19
*Min*	1.20	26.00	16.33	1.20	27.00	16.48
*Maks*.	1.55	53.00	25.20	1.57	55.00	27.78

**Table 3:** Anthropometric indicators among 11–12-year-old children depending on the occurrence of chest pain and gender.

**Children 11–12 years old**						
	**Girls from study group (** ***n*** **=** **15)**			**Boys from the study group (** ***n*** **=** **15)**		
	**height (m)**	**weight (kg)**	**BMI**	**height (m)**	**weight (kg)**	**BMI**
*M*	1.53	43.93	18.62	1.53	45.00	19.05
*SD*	0.08	5.87	1.43	0.12	10.86	2.64
*Me*	1.54	45.00	18.51	1.51	45.00	19.47
*Min*	1.35	31.00	16.65	1.40	30.00	13.85
*Maks*.	1.64	52.00	22.21	1.81	70.00	22.22
	**Girls from the control group (** ***n*** **=** **40)**			**Boys from the control group (** ***n*** **=** **33)**		
	**height (m)**	**weight (kg)**	**BMI**	**height (m)**	**weight (kg)**	**BMI**
*M*	1.42	39.80	19.73	1.37	37.58	19.98
*SD*	0.12	7.60	2.33	0.16	7.62	2.58
*Me*	1.44	39.00	19.44	1.40	37.00	19.17
*Min*	1.20	25.00	17.12	1.10	23.00	17.09
*Maks*.	1.60	55.00	25.44	1.64	53.00	29.24

IBM SPSS Statistics version 25 was used to conduct the study. Due to the observed deviations of the distribution of variables from the Gaussian curve, as well as considering the small size of the youngest group and different numbers in the groups of older children, non-parametric tests were used to analyze intergroup differences. We resolved to check whether the level of indicators of body posture characteristic for children from the test group differs significantly from the level of the same indicators in the children from the control group. Using the Mann-Whitney *U*-test, the results of the study group were compared with those of the control group. The significance level in this study was *p* = 0.05, while the scores of *p*-values in the range between 0.05 and 0.1 (0.05 < *p* < 0.1) were considered to indicate the significance of the test statistic at the level of the statistical tendency.

The achieved scores show that the children aged 7–8 years from the study group did not differ statistically significantly from their peers in terms of the trunk imbalance VP-DM (mm), pelvic skewness DL-DR (mm), pelvic torsion DL- DR [°], kyphosis angle VP-ITL [°] and lordosis angle ITL-DM [°], surface rotation (rms) [°] and lateral deviation VPDM (rms) (mm) (Table 4). The value of the effect size for the intergroup differences in the lateral deviation of VPDM (rms) (mm) suggests that an increase in the number of subjects in each subgroup would contribute to significant differences within this variable. The effect size would be moderate. According to the data in Table 4, the children from the study group would be characterized by a greater lateral deviation compared to children who do not struggle with pain.

**Table 4:** Mean scores, standard deviations and the significance of differences in indicators of body posture in children aged 7–8 years, depending on the appearance of pain.

	**Children from study group (** ***n*** **=** **14)**		**Children from control group (** ***n*** **=** **14)**				
	***M***	***SD***	***M***	***SD***	***U***	***p***	***R***
Trunk imbalanceVP-DM (mm)	6.25	4.70	5.91	3.65	97.00	0.963	0.01
Pelvic skewness DL-DR (mm)	2.09	2.17	1.89	2.17	94.00	0.845	0.04
Pelvic torsion DL-DR (°)	2.77	1.65	2.58	1.68	91.50	0.765	0.07
Kyphosis angle VP-ITL (°)	39.94	6.98	38.26	8.59	96.00	0.927	−0.02
Lordosis angle ITL-DM (°)	33.78	9.54	36.73	9.30	77.00	0.335	−0.21
Surface rotation (rms) (°)	4.13	1.71	3.81	1.66	86.00	0.581	0.12
Lateral deviation VPDM (rms) (mm)	4.08	2.97	2.57	1.24	63.00	0.108	0.36

The same calculations were made in the group of children aged 9–10 years. According to the data in Table 5, there were statistically significant differences between the analyzed groups (children from the study group vs. children from the control group) in terms of trunk imbalance VP-DM (mm), pelvic skewness DL-DR (mm) and lateral deviation VPDM (rms) (mm). The mean scores achieved in individual subgroups showed that children aged 9–10 years from the study group were characterized by a bigger intensity of abnormalities in the above-mentioned variables compared to their peers from the control group. The size of these differences is moderate for trunk imbalance VP-DM (mm) and pelvic skewness DL-DR (mm) and very high for lateral deviation VPDM (rms) (mm). In the case of the remaining indicators of body posture, no significant differences were found.

**Table 5:** Mean scores, standard deviations and the significance of differences in indicators of body posture in children aged 9–10 years, depending on the occurrence of chest pain.

	**Children from study group (** ***n*** **=** **20)**		**Children from control group (** ***n*** **=** **33)**				
	***M***	***SD***	***M***	***SD***	***U***	***p***	***r***
Trunk imbalance VP-DM (mm)	5.62	3.69	3.46	3.38	209.50	0.027	0.37
Pelvic skewness DL-DR (mm)	5.40	4.63	2.69	2.19	212.00	0.026	0.36
Pelvic torsion DL-DR [°]	2.77	2.17	1.88	1.40	267.00	0.248	0.19
Kyphosis angle VP-ITL [°]	42.26	7.45	38.90	7.52	242.00	0.106	0.27
Lordosis angle ITL-DM [°]	39.11	5.84	39.39	8.41	318.00	0.826	−0.04
Surface rotation (rms) [°]	4.28	1.83	4.03	2.03	298.00	0.557	0.10
Lateral deviation VPDM (rms) (mm)	4.25	1.38	2.17	0.89	70.00	0.001	0.79

## Discussion

It is assumed that each habitual and bad body posture affects the load on the locomotor system. This leads to disorders in the muscle balance, which in turn reduces the efficiency of anti-gravity muscles and poorer motor control. Shaping the spine and positioning the pelvis play an important role in maintaining a good body posture. (24). Daşkapan et al. believe that postural disorders often appear in children with chest pain. The abnormalities mainly concern the position of the spine as well as muscle tension and flexibility (25) Based on the conducted study, it can be noticed that in children from the study group, body posture disorders were visible in all age groups.

When assessing body posture, the value of lateral deflection, surface rotation and pelvic skewness play an important role in the image of the DIERS system. According to Harzman's findings, a posture defect is considered when the lateral deviation is <5 mm, the surface rotation is <5 degrees, and the oblique position of the pelvis exceeds 5 mm (26). Referring the achieved results to these criteria, it can be seen that in the 9–10 years age group the children met all the criteria for posture defects, while children in the remaining age groups in the study group confirmed the assumptions regarding lateral deviation and surface rotation.

Further analyzes were done in the group of older children (aged 11–12 years). As a result of the tests, it turned out that children from the study group achieved significantly higher results in the lateral deviation of VPDM (rms) (mm) compared to their peers from the control group. The size of these differences is moderate. The level of the other variables is comparable in both groups. The results of the analyzes are presented in Table 6.

**Table 6:** Mean scores, standard deviations and the significance of differences in body posture indicators in children aged 11–12 years, depending on the occurrence of chest pain.

	**Children from study group (** ***n*** **=** **30)**		**Children from control group (** ***n*** **=** **73)**				
	***M***	***SD***	***M***	***SD***	***U***	***p***	***r***
Trunk imbalance VP-DM (mm)	5.45	4.70	3.90	3.12	921.00	0.203	0.16
Pelvic skewness DL-DR (mm)	3.21	2.62	2.89	4.21	919.50	0.177	0.16
Pelvic torsion DL-DR [°]	2.26	1.51	2.09	2.06	900.50	0.158	0.18
Kyphosis angle VP-ITL [°]	41.68	8.32	40.27	8.22	1007.50	0.525	0.08
Lordosis angle ITL-DM [°]	40.69	12.98	38.82	8.93	1072.00	0.867	0.02
Surface rotation (rms) [°]	3.76	1.56	3.60	2.02	979.50	0.402	0.11
Lateral deviation VPDM (rms) (mm)	3.42	1.68	2.32	1.03	641.50	0.001	0.41

Comparing the study group with the control group, there is a statistically significant difference in lateral deviation VPDM (rms) (mm) (*p* = 0.001). The children from the 9–10 and 11–12 years study groups achieved higher scores than their peers from the control group. In the group of the youngest children, in terms of the lateral deviation VPDM (rms) (mm), an increase in the number of studied children would contribute to significant differences in this variable. It seems that this parameter may influence the development of functional chest pain.

According to the study by Nowotny et al., the appearance of low-degree lateral deviation in the developmental age may cause pain in later age (27).

Szczepanik points aut that the lateral deviation is a disorder of the spine axis associated with the asymmetry of muscle tone and the habitual adoption of incorrect body posture (28).

Both the bone and ligament system as well as the muscular system affect proper shaping of body posture. According to Linek et al., weakness as well as improper functioning of muscles may contribute to the formation of spine deformities (29).

In the study group, among children aged 9–10 years, there were also statistically significant abnormalities regarding trunk imbalance (*p* = 0.027) and pelvic skewness (*p* = 0.026) compared to the children of the same age in the control group. The children from the study group in all age groups achieved higher scores regarding the size of kyphosis angle VP-ITL [°], pelvic torsion DL-DR [°] and surface rotation (rms) [°] than the children from the control group. However, these differences were not statistically significant. This may be due to the fact that in the chosen age group, worsening of body posture is (routinely) observed in children.

According to Maciałczyk-Paprocka et al., Proszkowiec et al., Brzek et al., and Skawiński et al., between the ages of 7 and 12 years, there are visible posture abnormalities related to new school duties. It is believed that prolonged sitting at the desk, asymmetrical carrying of backpacks and a small amount of activity oriented on maintaining good posture contribute to the development of musculoskeletal disorders. The abnormalities mainly concern the curvature/shape of the spine, the asymmetry of the torso and the work of the muscles (20, 30–32).

Walicka-Cypruś et al. observed among 7-year-old children that carrying backpacks heavier than 10% of their body weight affected changes in the sagittal plane. The abnormalities concerned shallowing of the lumbar lordosis and a tendency to vertical positioning of the sacrum (33). Drzał-Grabiec et al. in their studies on the affect of carrying backpacks on body posture observed that symmetrical carrying caused negative changes in the sagittal plane, while asymmetric carrying resulted in worsening of posture in all planes (34).

According to the literature, postural disorders may also be associated with bad habits, sedentary lifestyle or the development of civilization aiming at technological progress (35, 36). Based on the research by Lastro et al. on the influence of a sedentary and active lifestyle on the body posture of children aged 10–16 years, fewer abnormal habits related to body posture were observed among children practicing sports than in sports inactive children (37). Both sedentary and inactive lifestyle among children is conducive to the appearance of back pain (38, 39). Frequent adoption of bad body posture affects not only the load on the spine structures, but also contributes to the development of disorders related to muscle tension (40–42). Some authors associate the development of chest pain with an incorrect position of the pelvis and a rounded back (4).

According to Biesemans et al. an increasing number of general practitioners recognize the need for new diagnostic tools to assess patients with chest pain (43).

According to Degenhardt et al., the DIERS formetric 4D system is an objective tool which can be used to assess the shape of the spine. The study conducted by the authors confirmed high efficiency of the DIERS system for the spine scan (44). Roman et al., in the studies on the correlation of the appearance of non-specific lower back pain with the shape of the spine, also emphasize the role of the DIERS formetric 4D system. Owing to the objective method of examining the body posture, the authors presented multidimensional analyzes which enabled the creation of an algorithm to predict functional disability for men and women (45).

## Conclusions

1. Irregularities in the parameters determining body posture may cause chest pain in children.
2. In the majority of children in the study group, the abnormalities of body posture were related to the lateral spine deviation.
3. The authors believe that prophylaxis of correct body posture can prevent chest pain amongchildren.
4. More research is needed to establish a rehabilitation program for children with chest pain.

## Limitations

All children diagnosed with chest pain during the project period in a large pediatric hospital were included in the study, but the number of analyzed cases may be too small to draw general conclusions. The presented topic requires further analyzes to define general indications in the described population. The response from the children is also one of the limitations of the study.

## Data Availability Statement

The raw data supporting the conclusions of this article will be made available by the authors, without undue reservation.

## Ethics Statement

The studies involving human participants were reviewed and approved by the study was approved by the Bioethics Committee at the Faculty of Medicine and Health Sciences, the Jan Kochanowski University in Kielce, Approval No. 46/2018, issued 18.09.2018. Written informed consent to participate in this study was provided by the participants' legal guardian/next of kin.

## Author Contributions

AZ, WK, and ZŚ contributed to conception and design of the study. AZ organized the database. GŚ performed the statistical analysis. AZ wrote the first draft of the manuscript. AZ, AŁŻ, GŚ, WK, and ZŚ wrote sections of the manuscript. All authors contributed to the article and approved the submitted version.

## Conflict of Interest

The authors declare that the research was conducted in the absence of any commercial or financial relationships that could be construed as a potential conflict of interest.

## Publisher's Note

All claims expressed in this article are solely those of the authors and do not necessarily represent those of their affiliated organizations, or those of the publisher, the editors and the reviewers. Any product that may be evaluated in this article, or claim that may be made by its manufacturer, is not guaranteed or endorsed by the publisher.
